# Analysis of DNA methylation in chondrocytes in rats with knee osteoarthritis

**DOI:** 10.1186/s12891-017-1739-2

**Published:** 2017-08-31

**Authors:** Xinxin Wang, Dezhi Tang, Peng Shen, Hao Xu, Hongfu Qiu, Tao Wu, Xiang Gao

**Affiliations:** 1 Department of Surgery, Huadong Hospital Affiliated to Fudan University, Shanghai, 200040 China; 2 Spine Research Institute, Shanghai University of Traditional Chinese Medicine, Shanghai, 200032 China

**Keywords:** Knee osteoarthritis, C/ebpα, Cdk2, Fas, Bak, Methylation

## Abstract

**Background:**

Knee osteoarthritis (KOA) is a degenerative knee disease commonly found in the ageing population. DNA methylation works with histone acetylation to participate in aging. Alterations of DNA methylation may involve the joint chondrocyte degeneration in KOA. The aim of this study is to detect DNA methylation changes in chondrocytes of rats with KOA.

**Methods:**

The rat KOA model was established with the Hulth method (*n* = 10), while rats receiving sham operation served as the control (*n* = 10). At 16 weeks after modeling, the knee joint tissue was collected from half of the rats in each group for Micro-CT scanning, Haematoxylin& Eosin (HE) staining, ABH/OG staining, immunohistochemistry for Bax, Bcl-2 and Fas, and TUNNEL staining. Meanwhile, the articular cartilage was collected from the other half to detect promoter methylation in target genes with the MethylTarget approach.

**Results:**

Micro-CT scanning, HE staining, ABH/OG staining, immunohistochemistry, and TUNNEL staining all showed more severe cartilage injury in the KOA group than in the control group, indicating successful establishment of KOA model. The methylation rate in the KOA group was significantly decreased for C/ebpα-2 (within a CpG island -452 bp to the initiation codon on chromosome 1 91,363,511), Cdk2 (within a CpG island -55 bp to the initiation codon on chromosome 7 3,132,362), Bak1 (within a CpG island 6452 bp to the initiation codon on chromosome 20 5,622,277), and Fas (within a CpG island on the entire chromosome 1 gene), compared with the sham group (*P* = 0.005, 0.008, 0.022 and 0.027, respectively).

**Conclusion:**

The chondrocyte apoptosis and significantly reduced methylation levels of C/ebpα-2, Cdk2, Bak1, and Fas may participate in the pathogenesis of KOA. However, the exact mechanisms remain to be determined.

## Background

Knee osteoarthritis (KOA) is a degenerative knee disease commonly found in the ageing population. It is usually derived from pathology of the cartilage, subchondral bone and synovial membrane of the knee joint. KOA is clinically characterized as progressive knee pain, swelling, stiffness, and restricted joint range of motion, and even joint deformity in serious conditions, resulting in loss-of-function of the joint, largely affecting the life quality of patients and posing a great economic burden [1]. Currently, there is no well treatment for KOA. All therapeutic approaches are aimed at ameliorating symptoms and improving joint functions. KOA is a multi-factorial disease. Apart from living style and healthy conditions, other factors like genetic aberrations and environmental factors also contribute to its occurrence. Changes in genetic modulation in cartilage have been implicated in the pathogenesis and progression of KOA. DNA methylation is one such player that has been receiving more and more attention. DNA methylation is a typical epigenetic modification of DNA to modify gene expression, which involves a process of transference of a methyl group on S-adenosyl methionine to the 5th atom of the cytosine ring under the catalysis effect of DNA methyltransferase, forming 5-methylcytosine. DNA methylation plays critical roles in embryo development, X chromosome inactivation, regulation of gene expression, gene imprinting and silencing, and tumorigenesis [2]. In addition, it works with histone acetylation to participate in aging. Alterations of DNA methylation may involve the joint chondrocyte degeneration in the elderly KOA patients. In this study, methylation was analyzed in KOA rats for 31 genes (C/ebpa, Cdk, Bak, Bax, Fas, Bcl, Tnfaip3, Tnfsf11, Il6r, Rel, Ikzf3, Irf5, Kdm4b, Aire, Nr3c1, Ptpn2, Mlh1, Irf8, Ifngr2, Nfkbie, Irak1, and Kdm6b, etc.) selected by searching the literatures [3–19]. These genes are directly or indirectly related to the metabolism of bone or cartilage through a common mechanism. By detecting the changes in methylation level of these genes, we hope to clarify the relationship between methylation alterations and KOA pathogenesis.

## Methods

### Animals

Twenty SPF-grade male SD rats (8 weeks, 180–200 g, Charles River Laboratories, Beijing, China) were randomly assigned into sham-operation and KOA model groups (*n* = 10 in each group). The rats were housed in groups of 5 in cages under room temperature of 21–22 °C with a 12 h light/dark cycle and free access to food and water. KOA modeling was performed after 1-week habituation. After fasting for 12 h, KOA model was established in the rats with the Hulth method [20]. Briefly, after anesthesia with intraperitoneal injection of 10% chloral hydrate, a longitudinal incision of 10 cm was made on skin at the inner side of the right hind knee of rat placed in a supine position. The medial collateral ligament was cut to open up the joint cavity, and medial meniscus was resected, then the anterior and posterior cruciate ligaments were cut. The anteriordrawer test was used to confirm rupture of the ligaments. Efforts were made to avoid artificial injury to articular cartilage. Sutures were made layer-by-layer. Rats in the sham group received only the same operation of skin incision. After surgery, chlortetracycline ointment was applied to the incision site and the surroundings to prevent infection.

### Sample preparation

At 16 weeks after modeling, rats in each group were sacrificed by injection with pentobarbital sodium at a dose of 0.2 ml/100 g. The articular cartilage was collected from 5 rats in each group and stored in formalin until use for methylation analysis. Meanwhile, the knee joint of the right hind limb was collected from the left rats, fixed in formalin for 48 h, and stored in 75% ethanol at room temperature until Micro-CT scanning, Haematoxylin& Eosin (HE) staining, orange G (ABH/OG) staining, immunohistochemistry and TUNNEL staining.

### Sample processing

After Micro-CT scanning (Scanco, vivaCT80), the knee joint tissue was decalcified in 14% EDTA (pH 7.40 ± 0.05) for 4 weeks with replacement of the decalcification medium every 2–3 days, followed by dehydration, wax immersing and embedding. Then the tissue was cut at 5 μm, and subsequently HE and ABH/OG staining, immunohistochemistry, and TUNNEL staining were conducted.

The articular cartilage underwent MethylTarget analysis of methylation of CpGIsland in the promoter region of 31 genes including C/ebpa, Cdk, Bak, Bax, Fas, Bcl, Tnfaip3, Tnfsf11, Il6r, Rel, Ikzf3, Irf5, Kdm4b, Aire, Nr3c1, Ptpn2, Mlh1, Irf8, Ifngr2, Nfkbie, Irak1, Kdm6b, etc. The MethylTarget test is an approach for DNA methylation level mapping following bisulfite treatment and bioinformatics data analysis, based on the second-generation high throughput sequencing platform [21, 22].

### Bisulfite conversion and multiplex amplification

DNA methylation level was analyzed by MethylTargetTM, an NGS-based multiple TargetedCpG methylation analysis method.Specifically, the genomic regions of interest were analyzed and transformed to bisulfite-converted sequences by geneCpG software. PCR primers (Table 1) were designed using the Methylation Primer software based on the bisulfate converted DNA.

**Table 1:** Primers of target genes

NO	Primer Name	sequence
1	Aire_F	ATAGTATTTAGATATTTAAGGGAGAAGGGAA
	Aire_R	ACCTAAAACTTATCCTCAAAAACCAC
2	Atm_F	GGTTTTATTGGYGGTGTTGAAT
	Atm_R	CACTCRCTCCCCTCAAAACATT
3	Bak1_1F	GTGGAYGAGAGTAGTTTTAAGTGGTG
	Bak1_1R	CATTTCTTCCCTAAACCATTAACC
	Bak1_2F	GGAGTTGATTTTTGTYGGGAGTT
	Bak1_2R	AACTCCTAACTACTCCCRAAACCAA
4	Bax_1F	GTATGYGTGAATTTAATGGTAGAGGTTT
	Bax_1R	TAATTCTCCRCCTCCCRCCTC
5	Bcl2_1F	GTTTTAGATTTTTAGGGGAGAGATATGTT
	Bcl2_1R	ACACAATACRCRAACTACTCCTTAAACAC
	Bcl2_2F	GGGYGTTAGGTGTAGTTGATTGGATA
	Bcl2_2R	CCTAACATCTTCTCCTTCCAACCT
6	Cdk2_2F	AAATTGTTAAGAGTTGAGTTTTGTTTG
	Cdk2_2R	TACCCTCTCCAATCTTCTCCAC
7	Cebpa_22F	GGATAGAAGGGGTTTGGTGAG
	Cebpa_22R	AACCATTACACTAAAAAATCTTAACCTTAC
	Cebpa_2F	TGGAAAGTTATAAGAGAAGGTAGGTTT
	Cebpa_2R	CCACCCAATACCCCAACTC
	Cebpa_3F	GTAAGGTTAAGAAGTYGGTGGATAAGA
	Cebpa_3R	CCTTAACCAAAAAACTCTCAAACAAC
	Cebpa_F	GGTTTTTTAGAGTAGTAGGGYGTTAGGA
	Cebpa_R	CCRAAACATTTAACTAAAAACTCCAC
8	Cflar_F	GGTTTTTTGAGATTTTTAGGGTTTATTAG
	Cflar_R	CTCCATTTTATAAACCCCAAAACA
9	Esr1_F	AAGATGTTTATGGAGAGGGTTTTG
	Esr1_R	AAAACCCCCAAACTATTAACACC
10	Fas_1F	GAGTTGTGTGGGTGTTAGTTTGTG
	Fas_1R	AACTACATATAAACATCTCTCATCACCAA
11	Icam1_F	TGGGGAGTTATTAAGAYGTTTTAGTAGTTA
	Icam1_R	CATTCAAAAATATCCCTCTCCCTA
12	ICOSLG_F	AGAGTGGAGAGTTGTAGTTGTTGG
	ICOSLG_R	CCCTACCCAACATCAAACTAAAC
13	Ifngr2_F	GTGGGTGTYGTTGGGAGTTT
	Ifngr2_R	CCTTCAACAAATCTCCCTAACA
14	Ikzf3_F	GGGTTGTAGTTGTTGTTTAGGTTT
	Ikzf3_R	CAAAATTCCTCAAACTTAACATTCAA
15	Il6r_F	GATTTAGGATGTAGTTGAGTAAGATTTGT
	Il6r_R	ACCAACCTAAAACTAACCCACCTATACT
16	Irak1_F	TGGATTTAGGTTTTTGGAGTTGA
	Irak1_R	CCCATCCCTAAAATCCCTAAAA
17	Irf5_F	GGAGTAGGGAGGATGTTTATTGG
	Irf5_R	AACTACTACCAAACCACCRCTCC
	Irf8_F	TAGTTGGGTTTTTTGGGAAAGTAA
	Irf8_R	ACCCCRCCCTATCTATAAAAACAAA
18	Kdm4b_2F	GGGTTGGTTGTGTGTATTTTGA
	Kdm4b_2R	CTCCCCTCATCAACACCTAAC
	Kdm4b_4F	AAGGGTGGAGTTYGGAGTATATAAGA
	Kdm4b_4R	TCAAAATACACACAACCAACCC
19	Kdm6b_2F	GAGTATATTTTAGGTTTTGGTAGGTAGGG
	Kdm6b_2R	CRCCAAAAACCCTTCTACCTTTAT
	Kdm6b_F	GGGAAGGTTAGGGAAGGGA
	Kdm6b_R	ACACRCATTTAACCAACACCCAC
20	Mlh1_1F	GTTGGGAAGGTGGTTTAGGA
	Mlh1_1R	CCAATTTTCAATCATCTCTTTAATAACA
	Mlh1_2F	GGTGTATTTTGTTYGGGTGATTTG
	Mlh1_2R	CRCAAACTCCACCACCAAATAAC
21	Nfkbie_F	GGGATTTAGGGTTTAGTGGGATT
	Nfkbie_R	AAAACCCTTCAACTACCCAAAA
22	Nr3c1_23F	GGTAGTTAGAGTTTTTGAGGGGATAGTT
	Nr3c1_23R	CTAACCCTCTCCTCTACRCTCCC
	Nr3c1_24F	AAGTTTGGGTTGTAGGTTGTGAG
	Nr3c1_24R	CRCCACCATCCCTAACCC
23	Nr3c1_F	GATTTTTAAGAGGTTAGGTAGAGGAGAT
	Nr3c1_R	CCCTCTACCTCATACCATAAACTAAA
24	Ppara_2F	GGTGTTAGGGTGGGTGTGG
	Ppara_2R	TACCCCCTACRCTATCAATTAACAA
	Ppara_F	TGAGGTGGGTGGATAGGG
	Ppara_R	CTCCAACCCACAAAACAACTAC
25	Ptpn2_F	GGAGGTTGTTGGTTTTGAAGG
	Ptpn2_R	CTAACCCCATCAAAAAACTCTCTC
26	Rel_F	GAATAGGTGTTTATTAGTAAGGGAAGG
	Rel_R	CAACCTTATTTTCCCTCTATATCTACATT
27	Tnfaip3_F	GGATGTGAYGTGGAAGGTAGTTTT
	Tnfaip3_R	AAAACTACAAACTAAACAATTCCCTTT
28	Tnfsf11_F	AGGAGATGGGTAGTTGTTTTGG
	Tnfsf11_R	AATCCTAAAACCAAACTCAATTTCC
29	Traf6_F	GGAGATATTTGATTTYGGAGTGTTG
	Traf6_R	CAACRACACRTCCTTATCCCTT
30	Tyk2_F	GATGATTAATAGGAATGTAGGATYGTG
	Tyk2_R	AACCTCCTCCCTACCAACTTC
31	Wnt2_F	TGAAGAGTTGATTTTAGGGGTGA
	Wnt2_R	AACTTTATCAATAAATCTCACCACTAACC

Genomic DNA (400 ng) was treated with sodium bisulfite using an EZ DNA Methylation™-GOLD Kit (Zymo Research) according to the manufacturer’s protocols. Multiplex PCR was performed with an optimized combination of primers. A 20 μl PCR reaction mixture was prepared for each reaction, which included 1× reaction buffer (Takara), 3 mM Mg2+, 0.2 mMdNTP, 0.1 μM of each primer, 1 U HotStarTaq polymerase (Takara) and 2 μl template DNA.The PCR program included 95 °C for 2 min; 11 cycles of 94 °C for 20 s, 63 °C for 40s with a decreasing temperature step of 0.5 °C per cycle, 72 °C for 1 min; then 24 cycles of 94 °C for 20 s, 65 °C for 30 s, 72 °C for 1 min; and finally 72 °C for 2 min.

### Index PCR

PCR amplicons were diluted and amplified using indexed primers. Specifically, a 20 μl mixture was prepared for each reaction, including 1× reaction buffer (NEB Q5TM), 0.3 mMdNTP, 0.3 μM F primer, 0.3 μM index primer, 1 U Q5TM DNA polymerase (NEB) and 1 μL diluted template. The PCR program was 98 °C for 30 s; 11 cycles of 98 °C for 10 s, 65 °C for 30 s, 72 °C for 30 s; and 72 °C for 5 min. PCR products (170 bp–270 bp) were separated by agarose electrophoresis and purified using QIAquick Gel Extraction kit (QIAGEN).

### Sequencing

Libraries from different samples were quantified, pooled together, and used for sequencing on the IlluminaMiSeq platform according to the manufacturer’s protocols. Sequencing was performed with a 2x300bp paired-end mode.

### Statistics

Quality control of sequencing reads was performed by FastQC. Filtered reads were mapped to genome by Blast after reads recalibration with USEARCH. All data are presented as mean ± SD and were analyzed with Independent samples t-test or U test to assess differences between groups. *P* < 0.05 was considered as statistically significant. Methylation and haplotype were analyzed using Perl script. Statistics were performed by t-test and ANOVA. Graphs were drawn with GraphPad Prism 5.0.

## Results

### Micro-CT, HE and ABH/OG staining

**Fig. 1:**
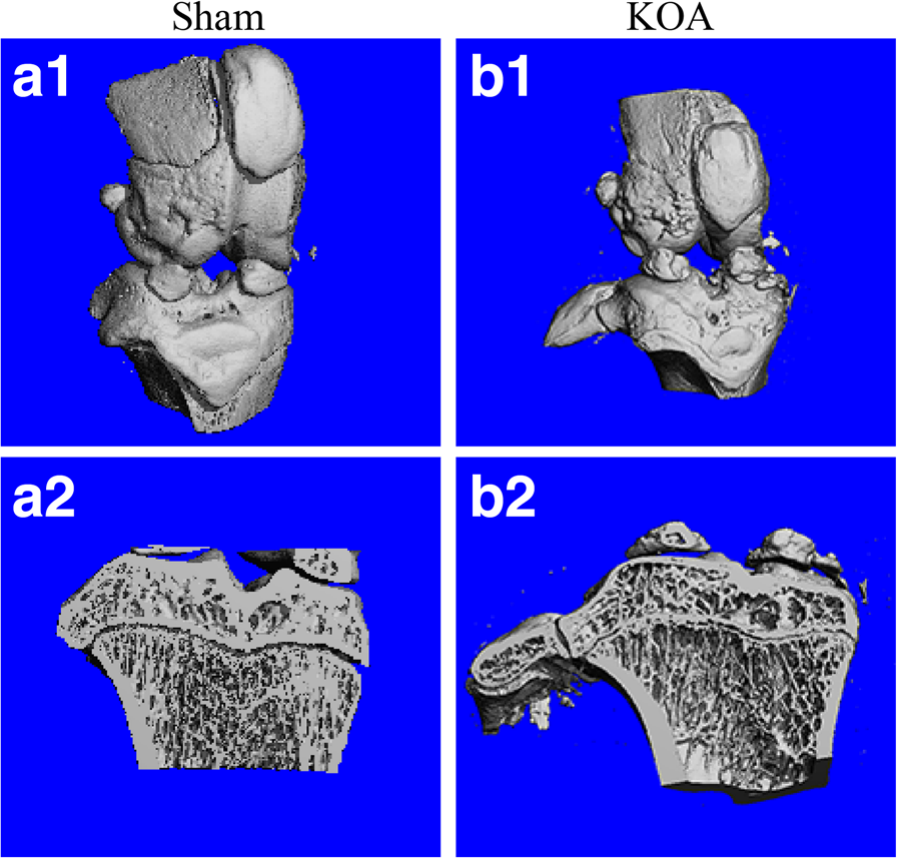
Images of Micro-CT scanning of the rat knee joint. **a1** and **b1** are reconstructed 3-dimensional images; **a2** and **b2** are images of coronal sections

The reconstructed 3-dimensional image of the articular cartilage by Micro-CT showed a smooth surface of the articular cartilage with no osteophyte formation in the sham group (Fig. 1a1). The coronal section image (Fig. 1a2) showed thick, uniformly aligned trabeculae. However, the KOA group showed cartilage defects such as a rough surface, focal surface fissures and flaking, and exposure of the subchondral bone (Fig. 1b1). Round or oval-shaped osteophyteswere formed along the lining of joint. The coronal section image (Fig. 1b2) showed a thin cartilage layer, small, disorganized trabeculae, and subchondral bone cystic degeneration.

**Fig. 2:**
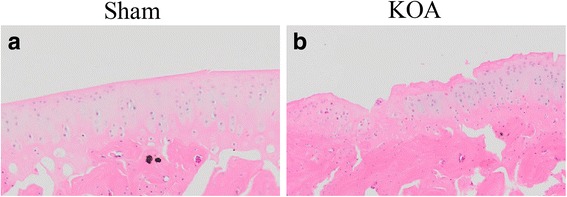
HE staining of the knee joint. **a**, sham group; **b**, KOA model group

**Fig. 3:**
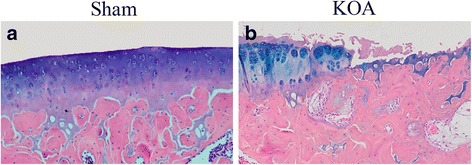
ABH/OG staining of the knee joint. **a**, sham group; **b**, KOA model group

HE staining revealed a smooth surface of cartilage in the sham group, with even coloration of cartilage matrix into light pink and blue chondrocyte nuclei (Fig. 2). The cartilage had clear structural layers and a tidal line. The out layer of cartilage was composed of flat chondrocytes, the middle layer composed of round uniformly-aligned chondrocytes, and inner and calcification layers composed of proliferating chondrocytes. Meanwhile, the cartilage matrix was evenly stained into blue by ABH/OG staining (Fig. 3). In the KOA model group, HE staining a thin cartilage layer, superficial fibrillation, necrosis of chondrocytes, unclear nucleus and cytoplasm, a rough surface with fissures and flaking, portional loss of cartilage, exposure of the subchondral bone, uneven coloration, unclear structural layers and tidal line, proliferation of chondrocytes in the outlayer, disordered cell alignment, appearance of cell clusters, and blood vessels passing through the tide line. Meanwhile, weak and negative staining was seen by ABH/OG staining, with clear fibrillation (Fig. 3). These results suggest the successful modeling of KOA in rats with the Hulth method.

### Immunohistochemistry and TUNNEL staining

**Fig. 4:**
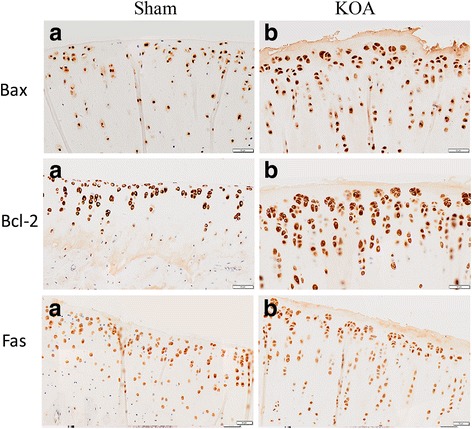
Immunohistochemistry of Bax, Bcl-2 and Fas in the knee joint. **a**, sham group; **b**, KOA model group

Immunohistochemistry for apoptotic proteins Bax, Bcl-2, and Fas revealed brown positive cells. Six view fields were randomly selected under a microscope to count the positive cells and calculate the positive rate. Results showed significantly increased levels of Bax, Bcl-2, and Fas in the KOA group than in the sham group (Fig. 4, Table 1). TUNNEL staining revealed a significantly higher apoptosis rate in the KOA group than in the sham group (Fig. 4, Table 2).

**Table 2:** Quantitative analysis of immunohistochemistry and TUNNEL staining

Staining	Positive rate (%)		*P* value
	Sham group	KOA group	
BAX	46.53 ± 3.86	88.62 ± 5.49	0.000
BCL-2	70.77 ± 7.61	85.99 ± 2.96	0.032
FAS	62.10 ± 1.68	89.77 ± 2.68	0.000
TUNNEL	50.65 ± 12.60	83.12 ± 13.79	0.040

### Methylation analysis

A significantly decreased methylation rate was detected in the KOA group forC/ebpα-2 (within a CpG island -452 bp to the initiation codon on chromosome 1 91,363,511), Cdk2 (within a CpG island -55 bp to the initiation codon on chromosome 73,132,362), Bak1 (within a CpG island 6452 bp to the initiation codon on chromosome 20 5,622,277), and Fas (within CpG islands on the entire chromosome 1 gene), compared with the sham group (Fig. 6, Table 3).

**Table 3:** Methylation rate in the promoter region of selected genes

Target genes (distance to the initiationcodon)	sham group	KOA group	*P* value
	Methylation rate (mean ± SD)		
C/ebpα-2 (−452 bp)	0.01096 ± 0.0043	0.002572 ± 0.0024	0.005
Cdk2 (−55 bp)	0.013573 ± 0.0052	0.004166 ± 0.0030	0.008
Bak1 (6452 bp)	0.026212 ± 0.0139	0.007123 ± 0.0058	0.022
Fas	0.958532 ± 0.0063	0.944658 ± 0.0096	0.047

## Discussion

KOA is a multifactorial disease with multiple contributors including genetic aberrations and environmental factors apart from life style and healthy conditions. Pathologically, KOA mainly involves degeneration of the cartilage and alterations of the cartilage matrix. Abnormalities of gene regulation in chondrocytes may be implicated in the pathogenesis and progression of KOA. DNA methylation is a typical form of epigenetic modification of DNA, and it works with histone acetylation to affect the aging process. Abnormal epigenetic modification is closely associated with KOA pathology as a result of environmental changes or aging, and affects KOA occurrence and progression via epigenetic modification. As epigenetic modification is a reversible process, it is reasonable to discover its role in KOA pathology, in which it slows down the development or even prevents the occurrence of KOA. In this study, we selected 31 KOA-related genes by searching literatures, and sequence their methylated sites, in order to clarify the relationship between gene methylation and KOA.

**Fig. 5:**
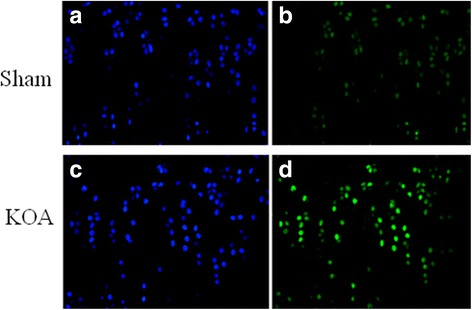
TUNEL staining of the knee joint. **a** and **c**, nuclei of chondrocyte; **b** and **d**, nuclei of apoptotic cells

**Fig. 6:**
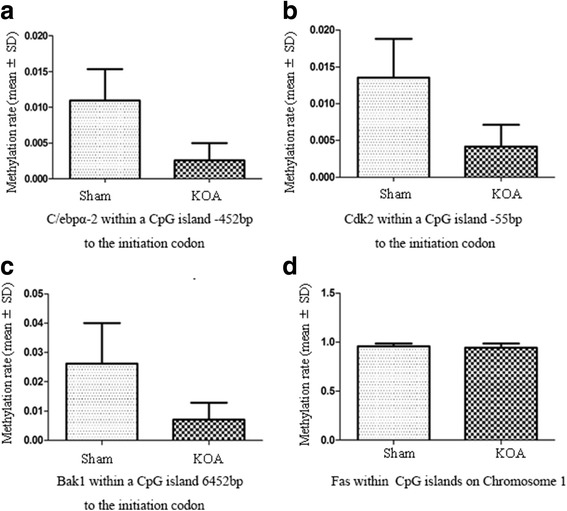
Methylation rate in the promoter region of C/ebpα (**a**), Cdk2 (**b**), Bak1 (**c**), and Fas (**d**)

Results of Micro-CT scanning (Fig. 1), HE staining (Fig. 2), ABH/OG staining (Fig. 3), immunohistochemistry (Fig. 4) and TUNNEL staining (Fig. 5) all confirmed the successful establishment of KOA model in rats. In addition, the methylation rate in the KOA group was significantly lower than that in the sham group for genes C/ebpα-2 (within a CpG island -452 bp to the initiation codon on chromosome 1 91,363,511), Cdk2 (within a CpG island -55 bp to the initiation codon on chromosome 7 3,132,362), Bak1 (within a CpG island 6452 bp to the initiation codon on chromosome 20 5,622,277), and Fas (within a CpG island on the entire chromosome 1 gene) (Fig. 6, Table 2). The decreased methylation in KOA chondrocytes may cause increased expression of these genes, which would further induce chondrocyte injury or apoptosis, leading to the pathogenesis and progression of KOA.

Aggravated chondrocyte injury or apoptosis occurs upon KOA onset [23]. Cartilage does not contain blood vessels. It has a low regeneration capacity and is therefore hard to repair after injury. When cartilage injury is so severe that the subchondral bone is damaged, rupture of small blood vessels would occur within the marrow cavity of cancellous bone. Then the blood forms a layer of fibrous clot on the surface of damaged cartilage area. In the absence of an external overload, the undifferentiated mesenchymal cells in bone marrow would move into this clot, whereby they differentiate into chondroblasts and proliferate into cells with chondrocyte morphology and typical features, in most cases fibrocartilage. Generally, an injury with a diameter < 3 mm can repair through generation of a hyaline cartilage, while a larger area of injury would repair by fibrous tissue [24]. Under normal conditions, C/ebpα can modulate the differentiation and proliferation of bone marrow mesenchymal stem cells into chondrocytes, and inhibit apoptosis of chondrocytes.

Here we found decreased methylation in promoter regions of C/ebpα and Cdk2 genes, which may induce overexpression of them. C/ebpɑ is present in multiple cell types, and participates in cell proliferation, differentiation, development, and tumorigenesis [25]. C/ebpα acts mainly to induce cell differentiation and inhibit cell proliferation [26]. The cyclin-dependent kinase Cdk2 can stimulate meiosis of germ cells and the G1-S transition during the mitotic cycle by interacting with cyclin E in early phases of DNA synthesis [27], thereby acting as a rate-limiting enzyme during cell proliferation [28]. Cdk2 activity peaks at S and G2 phases. In late phase of DNA replication, Cdk2 is activated by cyclin A2 to drive the S-G2 transition [29]. Wang H et al. found that C/ebpα directly interacts with Cdk2 to inhibit cell proliferation. In C/ebpα knockout cells, they found significantly enhanced activity of Cdk2, resulting in increased proliferation [30]. In this study, the decreased methylation level in promoter region of C/ebpα-2 gene in chondrocytes may cause overproduction of C/ebpα-2, thereby inhibiting Cdk2 activity and chondrocyte proliferation. However, we also detected a lower level of methylation in the promoter region of Cdk2 gene. This may be compensation to the inhibition of Cdk2 activity, which would increase Cdk2 expression to attenuate the inhibitory effect of C/ebpα-2.

It has also been reported that changes in the time of expression and activity of Cdk2 are associated with cell apoptosis [31]. The relative expression level of cyclins varies according to specific phases of cell cycle. Cyclin E is highly expressed in G1/S phase, while cyclin B is highly expressed in M phase in normal cells. However, in apoptotic cells cyclin B can also be detected in G1 phase, and likely, other cyclins can be detected out of their specific phases. This alteration may be associated with the overexpression of Cdks [32], implying that cyclins and Cdks have other functions than that in cell cycling in apoptotic cells. In this study, the decreased methylation of Cdk2 in chondrocytes may cause overexpression of Cdk2, inducing abnormal activation of cyclins and resulting in failure of mitosis and finally apoptosis. In fact, there is report that apoptosis is a form of mitosis failure [33].

The apoptosis of chondrocytes in KOA is also mediated by the Fas/FasL death receptor pathway or Bak-induced mitochondrial mechanism [34]. Fas binds to its ligand FasL to activate the tyrosine kinase, inducing phosphorylation of intracellular serine/threonine, finally leading to DNA cleavage and cell apoptosis [35, 36]. Fas can also induce cell apoptosis by indirectly activating triphosphate inositol and diglycerides, which then cause a rapid calcium release from the endoplasmic reticulum into cytoplasm and an influx of extracellular calcium, resulting in DNA breakage and chromosome shrinkage, and finally cell apoptosis [37]. Bak (Bcl-2 homologous antagonist/killer) is a member of Bcl-2 gene family, but with a contrast function of promoting cell apoptosis [36, 38, 39]. Machner et al. found that the expression of Fas mRNA in KOA patients was significantly higher than that in the control group by quantitative PCR [11]. Karaliotas et al. reported a notable overexpression of median mRNA levels of BAX was also observed in patients with stage III KOA compared with the control, while the BCL2/BAX ratio was markedlydecreased [40].Płucienniket al.found that the BCL2/BAX ratiomay regulate the gene promoter methylation [41]. Accumulating evidence has shown overproduction of pro-apoptotic factors like Fas and Bak in KOA chondrocytes, which causes increased cell apoptosis, while targeted inhibition of these factors reduces apoptosis [42–44]. Consistently, in this study we found increased expression of Fas, Bax and Bcl-2 in the KOA group than in the sham group using immunohistochemistry, and lower methylation levels in the promoter region of Fas and Bak1, which may induce their overexpression, thereby both leading to chondrocyte apoptosis. However, methylation in the promoter region of Fas genedid not play a key rolein the regulation at the level of its transcription, as the difference of the methylation of Fas gene between Sham and KOA groups was very slight. Li et al. reported that the transcription factors GA-binding protein (GABP) and activating protein-1 (AP-1) play a critical role in the induction of Fas mRNA [45]. Ryu et al.also found that Fas expression is transcriptionally induced by hypoxia-inducible factor (HIF)-2α [35]. In future, we will further study the transcriptional regulation of Fas gene by the transcription factors.

## Conclusion

To sum up, genetic aberrations are involved in KOA. Apoptosis of chondrocytes is a major pathological event in KOA pathogenesis and progression. Cartilage degeneration is attributed to multiple rather than a single risk factor. Findings in this study suggest that decreased methylation in promoter region of C/ebpα, Cdk2, Fas, and Bak1 participates in the pathogenesis of KOA, but the underlying mechanisms and pathways involved remain to be determined.

## Abbreviations

ABH/OG staining: Alcin blue/Orange G staining
Bak: Bcl-2 Homologous antagonist/killer
Bcl-2: B-cell lymphoma-2
C/ebpα: CCAAT/enhancer binding protein ɑ
Cdk: Cyclin dependent kinase
EDTA: Ethylene DiamineTetraacetic Acid
Fas: Factor associated suicide
HE staining: Haematoxylin & eosin staining
KOA: Knee osteoarthritis
TUNNEL: Terminal-deoxynucleotidyltransferase mediated nick end labeling
